# Sleep problems, decision-making, and suicide attempts during adolescence: a longitudinal birth cohort study

**DOI:** 10.1093/sleepadvances/zpaf062

**Published:** 2025-10-23

**Authors:** Michaela Pawley, Isabel Morales-Muñoz, Andrew P Bagshaw, Nicole K Y Tang

**Affiliations:** Department of Psychology, University of Warwick, Coventry, West Midlands, United Kingdom; School of Psychology, University of Birmingham, Birmingham, West Midlands, United Kingdom; Institute for Mental Health, University of Birmingham, Birmingham, West Midlands, United Kingdom; School of Psychology, University of Birmingham, Birmingham, West Midlands, United Kingdom; Centre for Human Brain Health, University of Birmingham, Birmingham, West Midlands, United Kingdom; Department of Psychology, University of Warwick, Coventry, West Midlands, United Kingdom

**Keywords:** sleep problems, suicide prevention, adolescents, longitudinal survey, decision-making

## Abstract

**Study Objectives:**

Sleep problems have been identified as a risk factor for suicidal thoughts and behaviors during adolescence, yet a lack of longitudinal mechanistic investigation into contributing factors (e.g. cognitive functioning) limits understanding of the temporality and specificity of this relationship. This study investigates the impact of sleep problems on subsequent reported suicide attempt, and whether risk-taking and decision-making moderate this relationship.

**Methods:**

This analysis utilized data from waves 6 (14 years) and 7 (17 years) in the Millennium Cohort Study (MCS; *n* = 8524, female = 4369 [51.26 per cent]), a UK population-representative longitudinal study of youth born between 2000 and 2002. Self-reported sleep items assessed at 14 years were used to calculate total time in bed on school and non-school nights, social jetlag, sleep onset latency, and frequency of night awakenings. Self-reported attempting suicide was measured at 17 years. The Cambridge Gambling Task assessed risk-taking and decision-making at 14 years.

**Results:**

Shorter total time in bed on school days (OR = 0.88; 95% CI = 0.80 to 0.96; *p* = .004) and more frequent night awakenings (OR = 1.15; 95% CI = 1.07 to 1.23; *p* ≤ .001) were prospectively associated with subsequent reported suicide attempt, even when controlling for demographic and clinical covariates. Rational decision-making (OR = 2.12; 95% CI = 1.33 to 3.37; *p* = .002) moderated the association between night awakenings and attempted suicide.

**Conclusions:**

Shorter total time in bed and more frequent night awakenings heightened suicide risk in adolescents, and this latter relationship was modified by rational decision-making. These results provide insight into the etiology of adolescent suicide behavior and highlight sleep deprivation and fragmentation as potential preventative targets for suicide attempts.

**Statement of Significance**

The development of adolescents coincides with the emergence of sleep problems and suicide-related behavior. Nevertheless, the influence of poor sleep on prospective adolescent suicide risk and relevant contributing factors remains unclear. This is the first longitudinal study examining the interrelationships between sleep, risk-taking and decision-making, and adolescent suicide attempts. Suicide attempt history at 17 years was associated with more frequent night awakenings and shorter total time in bed on school days at 14 years, and rational decision-making moderated this former association. These findings can inform suicide preventative interventions, highlighting the relevance of assessing and treating sleep deprivation and fragmentation. Future prospective studies utilizing multiple data waves should further examine the moderating mechanisms on the relationship between poor sleep and suicide risk.

## Graphical Abstract

**Graphical Abstract:**
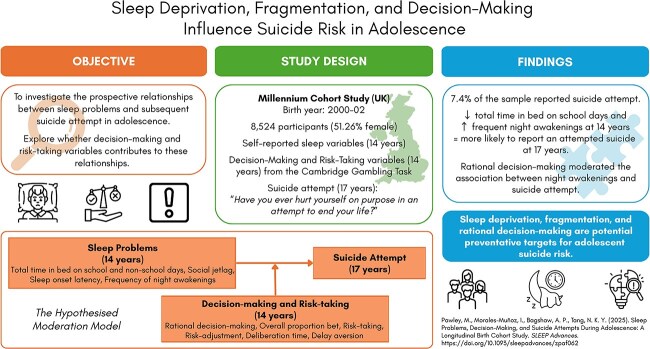

## Introduction

Adolescents often obtain insufficient sleep as a result of several biopsychosocial influences [1]. A circadian phase delay and reduced homeostatic sleep drive, as well as evening technology usage and early school start times, have contributed to this demographic experiencing later sleep timings and inadequate sleep [2]. Increased social jetlag (i.e. the discrepancy in timing between one’s chronotype and social obligations) is also common among adolescents [3] and is frequently countered with weekend catch-up sleep to compensate for the accumulated sleep debt [4]. Difficulty initiating and maintaining sleep [5], excessive daytime sleepiness [6], sleep variability between school and weekends [7], and poor sleep quality [5, 8] are also prevalent among this developmental age and have been associated with adverse outcomes, including poor mental health [9] and suicidal behavior [6, 10, 11].

Suicide is one of the leading causes of death among adolescents worldwide [12]. A 2020 meta-analysis estimated the global adolescent suicide rate at 3.77/100 000 [13], with upward trends detected in the United Kingdom, the United States, Central-Latin America, and Australasia [14]. Suicide attempts, which encompass one facet of the suicidal behavior continuum [15], are a major risk factor for completed suicide [16]. Therefore, research has strived to identify modifiable risk factors that contribute to attempting suicide to address the high rates of suicide in adolescents, with sleep emerging as a key influencer [17].

Longitudinal studies have demonstrated relationships between increased suicide risk in adolescence and insomnia [18], poor sleep quality [19, 20], short sleep duration [20, 21], and daytime sleepiness [21], although methods of sleep assessment vary, and findings have been inconsistent. Further research is needed to explore the predictive value of specific sleep parameters on longitudinal suicide risk during adolescence, to better understand the complex, multidimensional developmental trajectory of suicide behavior.

Despite research highlighting sleep as a prospective risk factor for adolescent suicide behaviors [7, 10, 11, 18–21], our understanding of the underlying mechanisms remains clouded. One potentially relevant factor is decision-making. For instance, adolescents typically report short sleep duration [22], which has been shown to contribute to risky decision-making [23]. Further, risk-taking [24] and decision-making [25, 26] have been identified as risk factors for attempting suicide among adults [27]. A 2021 meta-analysis that included 21 studies that utilized decision-making tasks found that adult suicide attempters demonstrate riskier decision-making compared to individuals with similar comorbid disorders but no suicide attempt history [28]. Similar results have been observed among young people [29].

Neuroimaging research has also noted reduced functional connectivity between the amygdala and the medial prefrontal cortex and increased functional connectivity between the amygdala and brain stem structures following one night of acute total sleep deprivation [30]. This may negatively impact attention and emotional processing [31], which are features fundamental to regulating risk-taking [32] and effective decision-making [33], as well as risk factors for suicidal thoughts and behaviors [34, 35]. With adolescence being marked by sleep problems [1], poor decision-making [36], increased risk-taking [37], and vulnerability to suicidal behavior [38], understanding the potentially intersected nature of these associations will provide insight into the developmental trajectory of suicidal behavior.

The present study seeks to explore the longitudinal association between multiple sleep variables (total time in bed on school and non-school days, social jetlag, sleep onset latency, and frequency of night awakenings) at 14 years old with the likelihood of reporting attempted suicide at 17 years old. It will also investigate the contributing role of risk-taking and decision-making in this association. Based on the existing body of literature, we hypothesized that a range of aspects of sleep disruption that are common in adolescence and also available in the chosen dataset, including greater social jetlag [39], longer sleep onset latency [18, 20], and more frequent night awakenings [18, 20], would be associated with a subsequent suicide attempt. Additionally, we expected total time in bed on school and non-school days to be related to suicide risk due to a key component of this variable, sleep duration, being linked with adolescent suicide behaviors [7, 20]. Furthermore, we predicted that risk-taking [24] and decision-making [26, 27] would play a moderating role in this relationship due to potentially amplifying or weakening the effect of poor sleep on suicide risk.

## Materials and Methods

### Sample

The Millennium Cohort Study (MCS) is an ongoing observational national birth cohort study that has been tracking the development of children born in the United Kingdom between November 2000 and January 2002 [40]. Seven waves of information have so far been collected when children were around 9 months (wave 1), 3 years (wave 2), 5 years (wave 3), 7 years (wave 4), 11 years (wave 5), 14 years (wave 6), and 17 years (wave 7). In the first wave, over 18 000 infants were recruited via a stratified cluster sampling designed to oversample young people living in disadvantaged areas [40]. Measurements have been collected on cohort members’ physical, cognitive, socio-emotional, and behavioral development, among other areas. Information has also been collected relating to daily life, behavior, relationships, economic circumstances, and attitudes.

The MCS was approved by the UK National Health Service Research Ethics Committee, and written consent was received from all participating parents at each survey until the cohort members were of age to give their own consent [41]. Data were downloaded from the UK Data Service, University of Essex, in December 2022, after registering with the UK Data Service.

For this study, we used data from ages 14 (wave 6) to 17 (wave 7) years. At 14 years old, 11 872 participants took part and at 17 years old, 10 757. Only the first child, or cohort member, surveyed in the household was included in the sample due to siblings from the same address holding the same identification number. This exclusion criterion aligns with other studies utilizing the MCS [42]. Our final sample for analysis consisted of 8524 participants who took part at both time points.

### Measures

#### Measures of self-reported sleep variables

The six items assessing sleep were developed by the MCS team [41] and self-reported at 14 years. These were: bedtime on (1) school and (2) non-school days, wakeup time on (3) school and (4) non-school days, (5) time taken to fall asleep over the past 4 weeks, and (6) the frequency of night awakenings during the last 4 weeks. These were adapted (see below) to generate five sleep variables for this study: (1) total time in bed on school nights, (2) total time in bed on non-school nights, (3) social jetlag, (4) sleep onset latency, and (5) frequency of night awakenings.

For bedtime and wake-up time on school and non-school nights, participants were asked: “About what time do you usually go to sleep on a school night/on the nights when you do not have school the next day?” and “About what time do you usually wake up in the morning on a school day/on the days when you do not have school?”. All response choices were recorded in accordance with prior research [43, 44]. Two variables for total time in bed were created by taking the difference between bedtime and wake-up time separately for school and non-school nights and were represented in hours. See Supplementary Material for more information on how variables were derived.

A measure of social jetlag was calculated by taking the difference between the midpoint of the sleep period for school nights and non-school nights [45]. The sleep midpoint was derived by computing the center between bedtime and wake-up time. This enabled the creation of a variable that represents misalignment in sleep timing between school and non-school days, measured in hours. Variation in sleep times between weekdays and weekends, approximating school and non-school days, has been shown to prospectively predict increases in suicide risk, beyond depressive symptoms [39].

Concerning sleep onset latency, participants were asked: “During the last four weeks, how long did it usually take for you to fall asleep?”. Our method of recoding the categorical response options (i.e. “0–15 min” [7.5 min], “16–30 min” [23 min], “31–45 min” [38 min], “46–60 min” [53 min], and “More than 60 min” [90 min]) into a continuous variable follows previous research utilizing this measure [43] to facilitate consistency in measurement and findings across studies utilizing the MCS, as well as with other sleep variables included within this study.

The frequency of night awakenings was assessed with the item: “During the last four weeks, how often did you awaken during your sleep time and have trouble falling back to sleep again?” Categorical response choices were inversely recoded from “1 = All of the time” (5), “2 = Most of the time” (4), “3 = A good bit of the time” (3), “4 = Some of the time” (2), “5 = A little of the time” (1), and “6 = None of the time” (0). This was done so higher values reflected more interrupted sleep, to be consistent with the other sleep measures in the logistic regression models.

#### Outcome measure of suicide attempt

Cohort members were asked about attempting suicide only at wave seven (17 years) through a self-completion questionnaire. Participants were asked “Have you ever hurt yourself on purpose in an attempt to end your life?” where responses were binary coded as “Yes” (1), and “No” (0).

#### Measures of risk-taking and decision-making

The Cambridge Gambling Task (CGT) [46] assesses dissociable aspects of decision-making and risk-taking behavior outside a learning context in both clinical and healthy populations [47]. Participants completed this measure on a computer screen in their homes during wave 6 (age 14 years). At the top of the screen were 10 red or blue boxes, with the ratio of colors varying from trial to trial. Participants were informed that a “token” was hidden behind one box. Each trial involved two choices. First, the participant selected which colored box (red or blue) the token was behind, then second, placed a bet of their points on their color decision. Bets were offered in an incrementally increasing or decreasing sequence in fixed proportions (between 5 per cent and 95 per cent) of their current points total. The ratio of blue to red boxes differed pseudo-randomly during the task to measure the effect of statistical probability on the quality of decision-making. Sampling participants’ gambling behaviors across various risk levels, the CGT offers outcome measures for risk-taking, risk adjustment, deliberation time, delay aversion, overall proportion bet, and rational decision-making.

This study assessed all outcome measures of the CGT to provide more thorough models of how risk-taking and decision-making may relate to suicidal behavior. See Supplementary Material for further information on each of these.

#### Covariates

Biopsychosocial covariates were selected based on prior research utilizing the MCS data [44, 48], as well as evidence supporting their influence on suicidal behavior: (1) sex (female/male) [49], (2) ethnicity (White [79.6 per cent]/other) [50], (3) socioeconomic status (low/high) [51], (4) early-onset tobacco use (yes/no) [52], (5) low self-esteem [53], (6) prior self-harm (yes/no) [54], (7) depressive symptoms [55], (8) peer problems [56], (9) emotional symptoms [56], (10) conduct problems [56], and (11) hyperactivity/inattention [56]. Depressive symptoms were measured using the Short Moods and Feelings Questionnaire [57] with greater values suggesting greater severity. Self-esteem was assessed with the Rosenberg Self-esteem Scale [58] with higher total scores indicating higher self-esteem. Peer problems, emotional symptoms, conduct problems, and hyperactivity/inattention were derived from the Strengths and Difficulties Questionnaire (SDQ) [59] with higher scores suggesting a higher level of psychological problems. All variables were obtained from wave 6 (14 years). See Supplementary Material for further details on ethnicity and socioeconomic status.

### Statistical analysis

Statistical analysis was performed using IBM SPSS Statistics (Version 29). The statistical analyses included descriptive analyses, correlations, logistic regression analyses, and interaction analyses. First, to assess for potential multicollinearity, all categorical predictors were converted into dummy variables and with the other predictor variables evaluated according to collinearity diagnostics in a regression model. The tolerance and variance inflation factor metric indicated that multicollinearity was not a concern [60]. Second, descriptive statistics for all predictor, moderator, and covariate variables were compared between participants who had reported attempted suicide and those without a suicide attempt history using the chi-square and independent *t*-tests. Third, Pearson correlations were computed between all sleep predictor variables, CGT measures, and continuous covariates to explore the initial associations. Fourth, multivariate logistic regression analyses were used to test the relationship between sleep variables at 14 years with reporting attempted suicide at 17 years. Two models were tested: an unadjusted model without covariates (model A), and an adjusted model (B). Model B included sex, ethnicity, family income, depression, self-esteem, prior self-harm, and regular smoking, and this was our primary model. Sensitivity analyses (model C) were conducted to assess the limits of the association between sleep at 14 years and subsequent suicide attempt history reported at 17 years in the presence of additional baseline mental health variables (emotional symptoms, conduct problems, hyperactivity/inattention, and peer problems). These were included alongside covariates from the primary model (B). Odds ratios (ORs) and 95% confidence intervals (CIs) were computed.

Finally, interaction models were tested to determine the moderating role of risk-taking and decision-making variables assessed by the CGT on the associations between sleep and suicide attempt. Significant sleep variables in our primary model (i.e. model B) were used in separate interaction analyses with the CGT variables to generate individual models. We opted for this stepwise analytic approach to ensure parsimony and reduce the risk of type I errors. Unadjusted and adjusted models were generated, using the same covariates from model B. Due to testing six models for each CGT variable, a Bonferroni-adjusted *p*-value was calculated to account for multiple testing by dividing the overall false positive rate by six [61].

#### Attrition and sampling weights

The final sample for this study included 8524 participants who had provided responses to all sleep items at 14 years and attempting suicide item at 17 years. As 46.7 per cent of the original sample was lost to attrition between the first and seventh wave, multiple logistic regressions were conducted to identify significant factors associated with drop-out as part of a missing data pattern analysis (see Table S1 in Supplementary Material for further information). Variables were selected based on their established relationship with systematic attrition bias and were predominately sociodemographic due to enhancing external validity in cohort studies [62]. These included birth weight, labor complications, ethnicity, sex, family income, having a longstanding illness, depressive symptoms, and having close friends at 14 years. Variables that were significantly associated with attrition were included in a logistic regression model (response vs nonresponse outcome) to generate propensity scores for each cohort member based on the conditional probability of participation at 17 years. These values were then multiplied by the marginal probability of participating at 17 years to generate stabilized inverse probability weights to correct for attrition biases, thereby enhancing the validity of causal inference [63]. Furthermore, the attrition weights were then multiplied by the MCS developed sampling weight, which accounts for unequal selection probabilities of wards [64], to ensure the final sample was representative of the target population.

## Results

Differences in sleep, socio-demographic, clinical, and decision-making and risk-taking variables between participants who had reported attempting suicide and those who did not report a suicide attempt are displayed in Table 1. All variables demonstrated significant differences between the two groups (*p* < .05). Specifically, the proportion of women, White ethnicity, low family income, regular smoking, and prior self-harm was higher among those who had attempted suicide. Additionally, the suicide attempt group also reported lower self-esteem, greater depressive symptoms, emotional symptoms, conduct problems, hyperactivity/inattention, and peer problems, shorter total time in bed on school and non-school days, higher social jetlag, longer sleep onset latency, more frequent night awakenings, lower rational decision-making, risk-taking, risk adjustment and overall proportion bet scores, and higher deliberation time and delay aversion than those without a history of suicide attempt. Further information on the frequency of these variables according to each wave can be found in the Supplementary Material.

**Table 1:** Characteristics of the sample according to attempted suicide

	Total (*n* = 9723)		Suicide attempt (*n* = 721)		No suicide attempt (*n* = 9002)		*t*/*X*^2^ value
*Sociodemographic variables*	*N* (%)		*N* (%)		*N* (%)		
Sex	9073		662 (7.3%)		8411 (92.7%)		
Male	4442 (49.0%)		192 (29.0%)		4250 (50.5%)		113.80^***^
Female	4631 (51.0%)		470 (71.0%)		4161 (49.5%)		
Ethnicity	8914		647 (7.3%)		8267 (92.7%)		
White	7047 (79.1%)		551 (85.2%)		6496 (78.6%)		15.71^***^
Other	1867 (20.9%)		96 (14.8%)		1771 (21.4%)		
Family income	9062		660 (7.3%)		8402 (92.7%)		
Low	2410 (26.6%)		250 (37.9%)		2160 (25.7%)		46.43^***^
High	6652 (73.4%)		410 (62.1%)		6242 (74.3%)		
*Psychological variables*	*N* (%)		*N* (%)		*N* (%)		
Regularly smokes cigarettes	8841		634 (7.2%)		8207 (92.8%)		
Yes	142 (1.6%)		34 (5.4%)		108 (1.3%)		60.99^***^
No	8699 (98.4%)		600 (94.6%)		8099 (98.7%)		
Self-harm	8850		632 (7.1%)		8218 (92.9%)		
Yes	1321 (14.9%)		354 (56.0%)		967 (11.8%)		904.75^***^
No	7529 (85.1%)		278 (44.0%)		7251 (88.2%)		
	Total *n*	Mean (SD)	Total *n*	Mean (SD)	Total *n*	Mean (SD)	
Self-esteem (score range 0–15)	8739	10.60 (2.90)	620	8.34 (3.56)	8139	10.77 (2.77)	16.66^***^
Depressive symptoms (score range 0–26)	8787	5.55 (5.85)	630	11.82 (7.78)	8157	5.07 (5.38)	−21.39^***^
Emotional symptoms (score range 0–10)	8816	2.00 (2.11)	642	3.28 (2.63)	8174	1.90 (2.03)	−13.02^***^
Conduct problems (score range 0–10)	8816	1.32 (1.56)	642	2.04 (1.91)	8172	1.26 (1.51)	−10.00^***^
Hyperactivity/inattention (score range 0–10)	8811	2.84 (2.34)	641	3.58 (2.64)	8170	2.79 (2.31)	−7.38^***^
Peer problems (score range 0–10)	8818	1.70 (1.80)	642	2.60 (2.13)	8176	1.63 (1.75)	−11.21^***^
*Sleep variables*	Total *n*	Mean (SD)	Total *n*	Mean (SD)	Total *n*	Mean (SD)	
Total time in bed school days (score range 5–13 h)	8956	8.64 (1.04)	650	8.21 (1.21)	8306	8.67 (1.02)	9.48^***^
Total time in bed non-school day (score range 7–16 h)	8950	10.53 (1.24)	649	10.40 (1.41)	8301	10.54 (1.22)	2.43^*^
Social jetlag (score range −2 to 5.5 h)	8944	1.96 (0.83)	649	2.04 (0.89)	8295	1.95 (0.83)	−2.68^**^
Sleep onset latency (score range 7.5–90 min)	8913	29.11 (24.16)	647	39.31 (29.42)	8266	28.32 (23.52)	−9.28^***^
Frequency of night awakenings (score range 0–5)	8940	1.37 (1.35)	648	2.18 (1.60)	8292	1.30 (1.30)	−13.65^***^
*Risk-taking and decision-making variables*	Total *n*	Mean (SD)	Total *n*	Mean (SD)	Total *n*	Mean (SD)	
Rational decision-making (score range 0.13–1.00)	8489	0.89 (0.13)	620	0.87 (0.14)	7869	0.89 (0.13)	2.82^**^
Risk-taking (score range 0.05–0.95)	8489	0.51 (0.15)	620	0.49 (0.15)	7869	0.52 (0.15)	3.58^***^
Risk adjustment (score range −3.86 to 5.10)	8489	1.03 (0.98)	620	0.93 (0.98)	7869	1.03 (0.97)	2.64^**^
Deliberation time (score range 362–23 691 ms)	8489	2328.79 (936.93)	620	2447.64 (1076.41)	7869	2319.43 (924.47)	−2.88^**^
Delay aversion (score range −0.90 to 0.90)	8486	0.27 (0.21)	620	0.28 (0.21)	7866	0.26 (0.21)	−2.12^**^
Overall proportion bet (score range 0.05–0.95)	8489	0.47 (0.14)	620	0.46 (0.14)	7869	0.47 (0.14)	3.12^**^

Descriptive and frequency information of the variables of interest, all of which were from 14 years (wave 6), in the Millennium Cohort Study according to reporting a suicide attempt at 17 years (wave 7). Group comparisons were generated between adolescents with and without a history of suicide attempt at 17. Categorical variables were compared using chi-square tests, and continuous variables were compared using independent sample *t*-tests.

Values are either *n* (%) or mean (standard deviation) corresponding with participants belonging to the group with a history of attempted suicide, no suicide attempt history, or all participants.

For depressive symptoms, emotional symptoms, conduct problems, hyperactivity/inattention, and peer problems, higher scores suggest a greater level of psychological problems or symptom severity. Higher self-esteem scores indicate higher self-esteem. Rational decision-making reflects the number of times the participant selected the most likely color the token was hidden behind; therefore, higher scores represent better rational decision-making. Risk-taking represents the average proportion of points the participant decided to risk betting on trials when they selected the most likely outcome, with higher scores indicating greater risk-taking. Risk adjustment indicates the tendency for the participant to increase their risk-taking when a larger proportion of the boxes are congruent with their selected color, thus higher scores indicate a proneness to bet higher when the odds of success are greater. Deliberation time is the average time taken to select the colored box which the token was hidden behind after being presented all boxes. Delay aversion represents participants tendency to bet larger amounts when bets are displayed in descending compared to ascending order, with higher scores suggesting betting behavior is more determined by presentation sequence than deliberation. Overall proportion bet reflects the average proportion of participant’s current points total bet across all trials, with higher scores indicating a tendency to take risks by gambling higher bets.

*t*, independent-samples *t*-test; *X*^2^, chi-square test.

Bold: *p* < .05.

^*^ *p* < .05; ^**^p < .01; ^***^p < .001.

Correlation analyses were conducted between all sleep variables and rational decision-making, risk-taking, risk adjustment, deliberation time, delay aversion, and overall proportion bet, as well as continuous covariates (see Table 2). These included self-esteem, depressive symptoms, emotional symptoms, conduct problems, hyperactivity/inattention, and peer problems. All correlations were relatively weak (*r* < .4) [65].

**Table 2:** Correlation analyses between sleep variables with decision-making and risk-taking variables and continuous covariates

	Total time in bed school	Total time in bed non-school	Social jetlag	Sleep onset latency	Night awakenings
	*r*	*r*	*r*	*r*	*r*
Rational decision-making	.02	−.02	−.05^***^	.00	−.07^***^
Delay aversion	.01	.01	.04^***^	.01	.04^***^
Deliberation time	.02^*^	.02^*^	.02^*^	.01	.02^*^
Overall proportion bet	.03^**^	.01	.01	.00	−.01
Risk adjustment	.01	−.04^***^	−.08^***^	−.02	−.08^***^
Risk-taking	.03^**^	.00	.00	.01	−.02
Self-esteem	.20^***^	.04^***^	−.07^***^	−.20^***^	−.27^***^
Depressive symptoms	−.26^***^	−.07^***^	.06^***^	.29^***^	.39^***^
Emotional symptoms	−.05^***^	−.00	.02	.12^***^	.20^***^
Conduct problems	−.07^***^	.01	.07^***^	.09^***^	.16^***^
Hyperactivity/Inattention	−.02	.00	.04^***^	.07^***^	.13^***^
Peer problems	−.01	.01	.00	.12^***^	.13^***^

Pearson correlations between all sleep predictor variables, Cambridge Gambling Task measures, and continuous covariates derived from 14 years (wave 5). Total time in bed on school days is significantly correlated with deliberation time, overall proportion bet, risk-taking, self-esteem, depressive symptoms, emotional symptoms, and conduct problems. Total time in bed on non-school days is significantly correlated with deliberation time, risk adjustment, self-esteem, and depressive symptoms. Social jetlag is significantly correlated with rational decision-making, delay aversion, deliberation time, risk adjustment, self-esteem, depressive symptoms, conduct problems, and hyperactivity/inattention. Sleep onset latency is significantly correlated with self-esteem, depressive symptoms, emotional symptoms, conduct problems, hyperactivity/inattention, and peer problems. Night awakenings are significantly correlated with rational decision-making, delay aversion, deliberation time, risk adjustment, self-esteem, depressive symptoms, emotional symptoms, conduct problems, hyperactivity/inattention, and peer problems.

*r*, Pearson correlation coefficient.

Bold: *p* < .05.

^*^ *p* < .05; ^**^p < .01; ^***^p < .001.

### Sleep and attempting suicide

The prospective relationships indicated by the logistic regressions between all sleep variables at 14 years and reporting of attempted suicide at 17 are found in Table 3. The multivariate unadjusted logistic regression model found shorter total time in bed on school days (OR = 0.72; 95% CI = 0.67 to 0.78; *p* ≤ .001), longer sleep onset latency (OR = 1.00; 95% CI = 1.00 to 1.01; *p* = .020), and more frequent night awakenings (OR = 1.45; 95% CI = 1.37 to 1.54; *p* ≤ .001) to significantly associate with an increased likelihood of reporting attempted suicide.

**Table 3:** Weighted unadjusted and adjusted associations between sleep variables at 14 and reported suicide attempt at 17

	Model A (*N* = 8524)			Model B (*N* = 8181)			Model C (*N* = 7955)		
	OR	95% CI	*P*-value	OR	95% CI	*P*-value	OR	95% CI	*P*-value
Total time in bed, school day	0.72	0.67, 0.78	<.001^***^	0.88	0.80, 0.96	.004^**^	0.84	0.77, 0.93	<.001^***^
Total time in bed, non-school day	0.98	0.91, 1.06	.639	0.97	0.89, 1.06	.516	0.97	0.88, 1.05	.435
Social jetlag	1.12	1.00, 1.26	.056	1.03	0.90, 1.18	.661	1.04	0.91, 1.19	.579
Sleep onset latency	1.00	1.00, 1.01	.020^*^	1.00	0.99, 1.00	.268	1.00	0.99, 1.00	.128
Night awakenings	1.45	1.37, 1.54	<.001^***^	1.15	1.07, 1.23	<.001^***^	1.12	1.04, 1.20	.002^**^
Sex	—	—	—	0.77	0.63, 0.94	.012^*^	0.65	0.52, 0.81	<.001^***^
Socioeconomic status	—	—	—	2.24	1.82, 2.76	<.001^***^	1.85	1.49, 2.30	<.001^***^
Ethnicity	—	—	—	1.43	1.07, 1.91	.017^*^	1.39	1.03, 1.87	.031^*^
Regularly smokes cigarettes, 14 years	—	—	—	1.00	0.63, 1.60	.997	0.79	0.49, 1.30	.357
Self-harm, 14 years	—	—	—	4.36	3.50, 5.42	<.001^***^	4.21	3.37, 5.26	<.001^***^
Self-esteem, 14 years	—	—	—	0.98	0.94, 1.02	.299	0.99	0.95, 1.03	.562
Depressive symptoms, 14 years	—	—	—	1.07	1.05, 1.09	<.001^***^	1.06	1.04, 1.08	<.001^***^
Emotional symptoms, 14 years	—	—	—	—	—	—	1.06	1.01, 1.11	.014^*^
Conduct problems, 14 years	—	—	—	—	—	—	1.08	1.01, 1.14	.025^*^
Hyperactivity/inattention, 14 years	—	—	—	—	—	—	1.05	1.00, 1.10	.069
Peer problems, 14 years	—	—	—	—	—	—	1.08	1.02, 1.14	.006^**^

Model A represents the unadjusted model, model B represents the primary adjusted model of the analysis, and model C displays the results from the sensitivity analysis. In model A, shorter total time in bed on school days, longer sleep onset latency, and more frequent night awakenings are significantly associated with reporting an attempted suicide. In the primary adjusted model (model B), shorter total time in bed on school days and more frequent night awakenings are significantly related to attempting suicide. In the sensitivity analysis (model C), shorter total time in bed on school days and more frequent night awakenings were significant predictors for reporting attempted suicide. In the adjusted models, variables were coded as = sex (female [referent] versus male), socioeconomic status (high [referent] vs. low), ethnicity (ethnic minority [referent] vs. White), regularly smokes cigarettes (no [referent] vs. yes), self-harm (no [referent] vs. yes), self-esteem (entered as a continuous variable with higher scores indicating good self-esteem), depressive symptoms (entered as a continuous variable with higher scores indicating greater severity), emotional symptoms, conduct problems, hyperactivity/inattention, and peer problems (entered as continuous variables with higher scores indicating higher levels of psychological problems).

Please note that the discrepancy in reported sample size with Table 1 is due to the multivariate logistic regression analysis only including participants that provided responses to all variables, while in Table 1, each variable was examined individually. Therefore, the sample size in Table 1 is greater than that reported in these models.

OR, odds ratio; 95% CI, 95% confidence interval.

Bold: *p* < .05.

^*^ *p* < .05; ^**^p < .01; ^***^p < .001.

In the primary model (model B), where biopsychosocial covariates at age 14 were included, only shorter total time in bed on school days (OR = 0.88; 95% CI = 0.80 to 0.96; *p* = .004) and more frequent night awakenings (OR = 1.15; 95% CI = 1.07 to 1.23; *p* ≤ .001) remained significant predictors for reporting a suicide attempt at 17. Several covariates were found to be significant confounding and risk factors, including being White (OR = 1.43; 95% CI = 1.07 to 1.91; *p* = .017), low socioeconomic status (OR = 2.24; 95% CI = 1.82 to 2.76; *p* ≤ .001), prior self-harm (OR = 4.36; 95% CI = 3.50 to 5.42; *p* ≤ .001), and depressive symptoms (OR = 1.07; 95% CI = 1.05 to 1.09; *p* ≤ .001). Identifying as male (OR = 0.77; 95% CI = 0.63 to 0.94; *p* = .012) was associated with a lower likelihood of attempting suicide.

### Sensitivity analysis

All sleep variables were included within the sensitivity analysis. After accounting for a range of additional psychosocial difficulties (see model C in Table 3), the same sleep variables that were associated with reporting attempted suicide in the primary model, namely, shorter total time in bed on school days (OR = 0.84; 95% CI = 0.77 to 0.93; *p* ≤ .001) and more frequent night awakenings (OR = 1.12; 95% CI = 1.04 to 1.20; *p* = .002), remained significant.

### The moderating role of risk-taking and decision-making between sleep and attempting suicide

As total time in bed on school days and night awakenings at age 14 were significantly associated with reporting a suicide attempt at age 17 in the primary logistic regression model, the interaction model with risk-taking and decision-making variables was only applied for these sleep variables (see Tables 4 and 5).

**Table 4:** Weighted unadjusted and adjusted associations between frequency of night awakenings and CGT variables at 14 years with reported attempted suicide at 17 years as the outcome

	Model A			Model B		
	OR	95% CI	*P*-value	OR	OR 95% CI	*P*-value
Night Awakenings × RDM	1.98	1.35, 2.90	<.001^***^	2.12	1.33, 3.37	.002^**^
Night awakenings	0.86	0.61, 1.20	.375	0.59	0.39, 0.90	.013^*^
RDM	0.11	0.04, 0.30	<.001^**^	0.09	0.03, 0.28	<.001^**^
Night Awakenings × DA	0.86	0.66, 1.11	.248	0.90	0.67, 1.21	.488
Night awakenings	1.63	1.49, 1.79	<.001^**^	1.18	1.06, 1.32	.002^**^
DA	1.70	0.84, 3.42	.140	1.45	0.69, 3.07	.330
Night Awakenings × DT	1.00	1.00, 1.00	.087	1.00	1.00, 1.00	.077
Night awakenings	1.76	1.52, 2.03	<.001^**^	1.32	1.12, 1.55	.001^**^
DT	1.00	1.00, 1.00	<.001^**^	1.00	1.00, 1.00	<.001^**^
Night Awakenings × OPB	0.86	0.58, 1.26	.431	0.95	0.61, 1.47	.812
Night awakenings	1.68	1.40, 2.02	<.001^**^	1.18	0.96, 1.45	.127
OPB	0.41	0.15, 1.13	.085	0.48	0.16, 1.46	.192
Night Awakenings × RA	1.08	1.02, 1.14	.010^*^	1.08	1.01, 1.15	.025^*^
Night awakenings	1.45	1.35, 1.56	<.001^**^	1.07	0.97, 1.17	.176
RA	0.77	0.67, 0.89	<.001^**^	0.80	0.68, 0.94	.007
Night Awakenings × RT	0.94	0.66, 1.36	.756	0.99	0.66, 1.50	.978
Night awakenings	1.61	1.33, 1.94	<.001^**^	1.15	0.93, 1.43	.193
RT	0.32	0.12, 0.84	.021^*^	0.44	0.15, 1.26	.124

Moderation analyses with frequency of night awakenings at 14 years as a predictor, each outcome measure from the Cambridge Gambling Task assessed at 14 years as a moderator and reporting an attempted suicide at 17 years as the dependent variable. After applying the Bonferroni correction for multiple testing, rational decision-making was found to significantly moderate the association between night awakenings and reported suicide attempt in both the unadjusted and adjusted models.

OR, odds ratio; 95% CI, 95% confidence interval; RDM, rational decision-making; DA, delay aversion; DT, deliberation time; OPB, overall proportion bet; RA, risk adjustment; RT, risk-taking.

Bold: *p* < .008.

^*^ *p* < .05; ^**^p < .008, Bonferroni corrected for the number of statistical tests undertaken in the table.

**Table 5:** Weighted unadjusted and adjusted associations between total time in bed on school days and CGT variables at 14 years with reported attempted suicide at 17 years as the outcome

	Model A			Model B		
	OR	95% CI	*P*-value	OR	OR 95% CI	*P*-value
Total Time in Bed School × RDM	0.98	0.57, 1.70	.944	1.35	0.70, 2.60	.372
Total time in bed school	0.65	0.40, 1.05	.080	0.66	0.37, 1.18	.164
RDM	0.45	0.01, 42.27	.732	0.03	0.00, 7.43	.214
Total Time in Bed School × DA	0.88	0.61, 1.27	.497	0.87	0.58, 1.30	.493
Total time in bed school	0.66	0.58, 0.75	<.001^**^	0.89	1.78, 1.02	.099
DA	4.28	0.20, 91.15	.352	4.01	0.14, 116.81	.419
Total Time in Bed School × DT	1.00	1.00, 1.00	.222	1.00	1.00, 1.00	.381
Total Time in Bed School	0.58	0.48, 0.69	<.001^**^	0.93	0.76, 1.15	.500
DT	1.00	1.00, 1.00	.487	1.00	1.00, 1.00	.160
Total Time in Bed School × OPB	1.89	1.09, 3.28	.023^*^	1.72	0.94, 3.13	.078
Total time in bed school	0.47	0.37, 0.62	<.001^**^	0.67	0.50, 0.90	.007^**^
OPB	0.00	0.00, 0.15	.006	0.01	0.00, 0.79	.040
Total Time in Bed School × RA	0.96	0.89, 1.04	.328	0.98	0.89, 1.08	.658
Total Time in Bed School	0.66	0.60, 0.74	<.001^**^	0.88	0.77, 0.99	.033^*^
RA	1.20	0.61, 2.36	.595	1.10	0.50, 2.41	.819
Total Time in Bed School × RT	1.69	1.01, 2.83	.046^*^	1.59	0.91, 2.80	.106
Total time in bed school	0.49	0.38, 0.64	<.001^**^	0.68	0.51, 0.92	.011^*^
RT	0.00	0.00, 0.27	.011^*^	0.01	0.00, 1.07	.053

Moderation analyses with total time in bed on school days at 14 years as a predictor, each outcome measure from the Cambridge Gambling Task assessed at 14 years as a moderator and reported attempted suicide at 17 years as the dependent variable. After applying the Bonferroni correction for multiple testing, no significant interactions were observed in any of the unadjusted or adjusted models.

OR, odds ratio; 95% CI, 95% confidence interval; RDM, rational decision-making; DA, delay aversion; DT, deliberation time; OPB, overall proportion bet; RA, risk adjustment; RT, risk-taking.

Bold: *p* < .008.

^*^ *p* < .05; ^**^p < .008, Bonferroni corrected for the number of statistical tests undertaken in the table.

**Figure 1:**
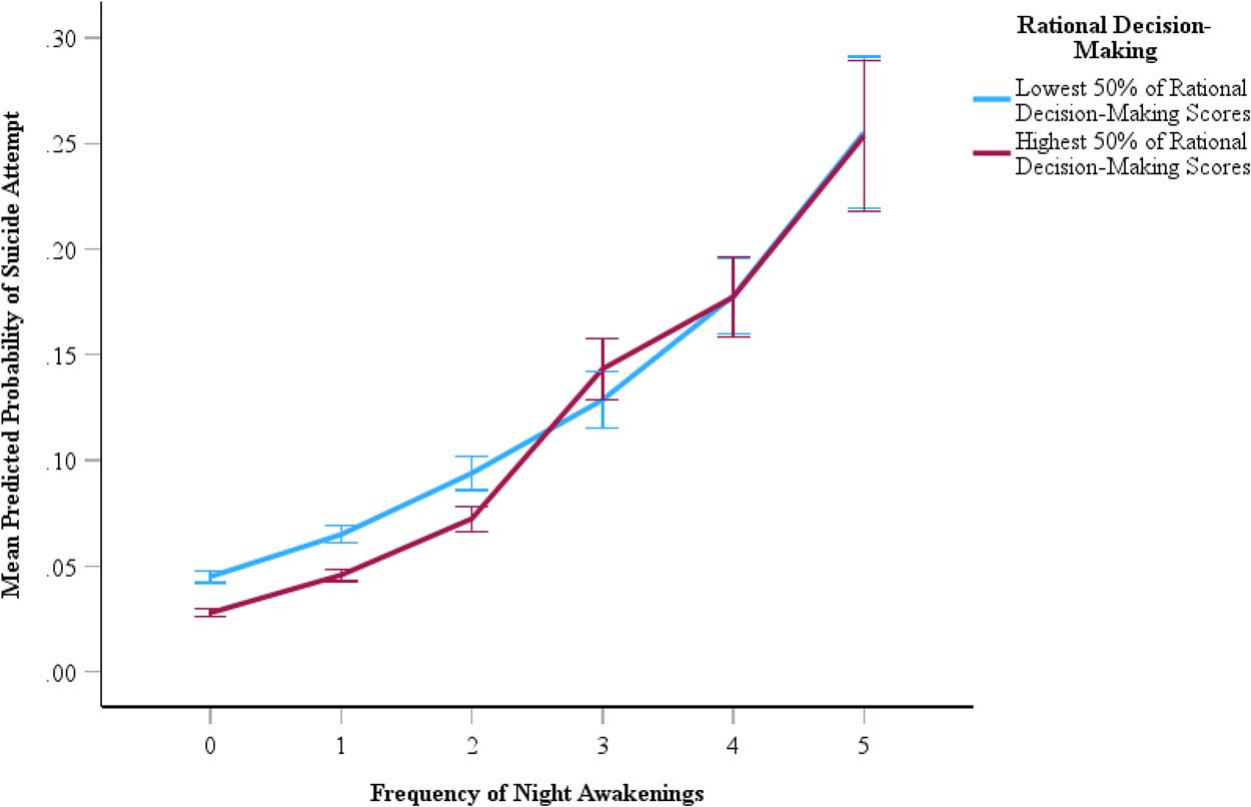
Associations between self-reported frequency of night awakenings at 14 years and the mean predicted probability of reporting an attempted suicide at 17 years at low (lowest 50 per cent of scores) and high (highest 50 per cent of scores) levels of rational decision-making. The blue line represents the 50 per cent of participants with the lowest rational decision-making scores and the red line reflects the remaining 50 per cent of participants with the highest rational decision-making scores. For both rational decision-making groups, an increase in night awakenings corresponds with an increased probability of reporting a suicide attempt. At low levels of night awakenings, those with high levels of rational decision-making have a slightly lower risk of subsequent reported suicide attempt compared to participants with low levels of rational decision-making. As the frequency of night awakenings increases, high and low levels of rational decision-making demonstrate similar risk for reporting a suicide attempt.

With frequency of night awakenings as a predictor, rational decision-making (OR = 2.12; 95% CI = 1.33 to 3.37; *p =* .002; see Figure 1) was found to significantly contribute to the association in the adjusted model (Bonferroni-adjusted *p* < .008 for each). For illustrative purposes, rational decision-making was dichotomized at the median to assess participants with low versus high levels. Participants with higher rational decision-making scores and fewer night awakenings had a slightly lower probability of attempting suicide compared to participants with lower rational decision-making. As the number of night awakenings increased, the buffering effect of rational decision-making was less pronounced. In the moderation analyses with total time in bed on school days as a predictor, no significant interactions were present in any of the unadjusted or adjusted models (Bonferroni-adjusted *p* < .008 for each). In all interaction models, low socioeconomic status, prior self-harm, and depressive symptoms were significant risk factors for later reporting of suicide attempts (see Supplementary Tables S3 and S4).

## Discussion

This study addressed the need for longitudinal research to establish the temporal association between poor sleep and suicidal behaviors in young people, with a secondary focus on the potential moderating roles of risk-taking and decision-making. Our main finding was that shorter total time in bed on school days and more frequent night awakenings at 14 years of age were associated with an increased likelihood of reporting attempted suicide at 17 years, over and above a range of other relevant biopsychosocial risk factors. Additionally, rational decision-making demonstrated a modulatory influence between night awakenings and the likelihood of attempting suicide, which, to the best of our knowledge, has yet to be explored in studies investigating the association between sleep and other types of suicide behavior or self-harm. These results complement and add to the existing research highlighting the importance of sleep deprivation and fragmentation, as well as decision-making on suicidal behavior among youth [6, 10, 11].

Total time in bed on school days and frequency of night awakenings demonstrated a strong association with subsequently reporting attempting suicide in adolescents, even when accounting for clinically relevant biopsychosocial factors, highlighting their significance as suicide risk factors. Additionally, several covariates were shown to predict suicide attempt disclosure beyond our significant sleep predictor and decision-making interaction. In the interaction model with night awakenings and rational decision-making, lower socioeconomic status [51] and prior self-harm [54] demonstrated a stronger effect on suicidality, which has also been supported by prior research. Nevertheless, when further testing the association between sleep and attempting suicide by including additional mental health variables in the sensitivity analysis, the effect of shorter total time in bed on school days and more frequent night awakenings as risk factors was stronger than depressive symptoms and the included psychosocial variables, which are all established suicide risk factors [55, 56]. This suggests that these biopsychosocial variables not only play a critical role in adolescent suicide risk but also stress the importance of specific sleep problems as additional risk factors.

### Total time in bed and night awakenings

Total time in bed on school days and frequency of night awakenings were the only sleep predictor variables associated with later reporting of suicidal behavior in the primary adjusted model.

Adolescents who reported a suicide attempt at 17 years were more likely to have reported shorter total time in bed on school days at 14 years. This corroborates previous cross-sectional [66–69] and longitudinal [70] findings that have found shorter sleep duration on school days to positively associate with suicide attempts in adolescents. Interestingly, Guo and colleagues [69] found the cross-sectional association between school day sleep duration and attempting suicide to be U-shaped, suggesting both short (5–7 h) and long (≥9 h) sleep duration can have negative implications on suicidality in youth. Nevertheless, this has not been demonstrated in other longitudinal studies [70]. Additionally, sex differences in the association between sleep duration and suicide attempt at 2-year follow-up have been noted, whereby only males with short sleep duration were at increased risk of suicidality [71]. It is important to note that this study investigated average sleep duration; thereby, differences in weekday versus weekend sleep duration on outcomes were not specified. Future longitudinal studies are necessary to determine whether there are sex or other sociodemographic differences in the association between weekday or weekend sleep duration and suicide attempts.

More frequent night awakenings at age 14 were positively associated with reporting an attempted suicide at the 3-year follow-up. Previous cross-sectional studies have similarly found sleep fragmentation to associate with suicide attempts among adolescents in clinical samples with depressive disorders and suicidal behaviors [72–74] and population-based samples [18]. Furthermore, our study extends the previously noted prospective effect of night awakenings on adolescent suicide attempt at 6-month follow-up [75]. The effect of sleep fragmentation on attempting suicide may be related to dysregulated rapid eye movement (REM) sleep, which can hinder emotion regulation functioning [76]. Given that REM sleep occurs after approximately 90 minutes of consolidated sleep and increases with each sleep stage [77], sleep disturbance through frequent night awakenings or sleep deprivation may disrupt one’s sleep architecture. This can contribute to difficulties in regulating one’s emotions, which has been associated with suicidal behavior [78] and may partially account for total time in bed on school days and night awakenings’ unique relationship with subsequent reported suicide attempt. Polysomnography or actigraphy research will be necessary to determine the extent to which sleep fragmentation and deprivation influence sleep architecture and subsequent suicidality.

### Decision-making and risk-taking as moderating factors

Adolescence is a period marked by vulnerability to poor, risky decisions [37], with the ability to engage in flexible goal-directed decision-making maturing as adolescents age [36]. Prior research has found associations between suicide attempters and risky decision-making [28, 29] and highlighted the importance of sleep in reducing risk-taking propensity [79] and promoting adaptive decision-making [80, 81]. To our knowledge, this study is the first to explore the longitudinal associations between sleep, risk-taking, decision-making, and reporting an attempted suicide during adolescence. Rational decision-making moderated the association between night awakenings and suicide attempt.

To prevent suicidal behavior, it is critical to understand whether and how decision-making influences one’s calculation of risk within a sleep context. Suicidal and self-harm behaviors each carry varying degrees of risk dependent on intention to die [82]. Rational decision-making reflects the tendency to make the optimal choice through selecting the most likely outcome in the CGT [46]. The moderating role of rational decision-making suggests that at lower levels of night awakenings, those with higher decision-making scores were less likely to report an attempted suicide compared to adolescents with lower decision-making scores, although this group difference narrowed with more frequent night awakenings. This suggests that the protective buffer of rational decision-making weakens as sleep fragmentation becomes more frequent, and other types of executive dysfunction, or even emotion regulation, may implicate this relationship [83]. Further research may benefit from exploring the influence of rational decision-making in relation to other psychological factors to determine which resources place individuals with sleep problems at greatest suicide risk.

Although our results indicated that the remaining decision-making and risk-taking measures assessed with the CGT did not significantly contribute to the association between sleep and suicide risk in this sample (i.e. delay aversion, deliberation time, overall proportion bet, risk adjustment, and risk-taking), other types of executive dysfunction, or even emotion dysregulation, may underlie this relationship [83]. Additionally, adolescence is characterised by dynamic maturational changes across biopsychosocial and contextual domains [84, 85] which may not be captured over such long intervals (i.e. 3 years between risk-taking and decision-making and reported suicide attempt). Therefore, future research may benefit from utilizing shorter timescales to capture this within-individual variability.

### Strengths and limitations

This study has several strengths, such as the large, representative sample size and inclusion of multiple sleep measures, adopting a longitudinal design to identify temporal associations, utilizing a neurocognitive assessment for decision-making and risk-taking, and incorporating several relevant biopsychosocial variables into the model. Nevertheless, findings should be considered within the limitations of the study.

First, self-reported sleep measures were single items, which can be susceptible to response bias and impair the predictive validity [86]. Furthermore, our derived measure for total time in bed from calculating the difference between bed and wake-up time impedes providing a direct or precise assessment. Additionally, night awakenings and sleep onset latency items addressed sleep complaints within the previous 4 weeks, thus reducing their specificity. Second, although this study adopted stabilized inverse probability weighting to account for attrition, there is debate over which weighting method is methodologically superior [87]. Future studies employing other weighting methods, like multiple imputation, in conjunction with sample weights, may further test the robustness of findings. Third, based on data available, the temporal association between sleep and reporting attempted suicide was restricted to 2–3 years. Previous research has adopted varying follow-up intervals between sleep problems and suicidal behavior; however, due to methodological constraints, such as variations in measurement, the use of single-item measures, and reliance on retrospective self-reporting, little improvement in predictive accuracy has been observed [88]. Furthermore, the question on suicide attempt did not specify when this behavior occurred; thus, associations found are in relation to the odds of reporting attempted suicide at 17 years rather than an actual subsequent suicide attempt. Exploring sleep phenotypes using validated measures in relation to other relevant factors and their influence on temporally specified suicide risk repeatedly throughout adolescence will provide greater insight into this detrimental developmental trajectory. Lastly, while the CGT is valuable in assessing multiple dissociable aspects of risk-taking and decision-making, the applicability of findings from decision-making tasks to suicidal behavior has yet to be established [89]. Longitudinal cohort studies typically explore a broad range of factors; thus, while employing a general decision-making task is practical, further research examining decision-making and risk-taking using tasks that are suicide-behavior specific is needed.

In summary, adolescents who experience difficulties maintaining and obtaining sufficient sleep appear to be more likely to report a suicide attempt several years later. Furthermore, rational decision-making moderated the relationship between night awakenings and attempting suicide. This provides a potential basis for identifying adolescents most at risk of the poorest outcomes and highlights shorter total time in bed on school days, frequent night awakenings, and decision-making as targets for interventions to reduce suicide risk.

## Supplementary Material

MPawley_SleepSuicideAttempt_SuppMaterial_FINAL_zpaf062

## Data Availability

The MCS datasets analyzed in this study can be found and accessed via the UK Data Service: https://discover.ukdataservice.ac.uk/series/?sn=2000031. Anyone wishing to use the MCS data must register and submit a data request to the UK Data Service at http://ukdataservice.ac.uk/. Analysis code is available from https://osf.io/rn8e9/.
