# Supplementary material for: Sleep problems, decision-making, and suicide attempts during adolescence: a longitudinal birth cohort study
Source: Sleep Adv. 2025 Oct 23;6(4):zpaf062. doi: 10.1093/sleepadvances/zpaf062 (PMC12546579; doi:10.1093/sleepadvances/zpaf062)
Supplement: MPawley_SleepSuicideAttempt_SuppMaterial_FINAL_zpaf062 [file mpawley_sleepsuicideattempt_suppmaterial_final_zpaf062.docx]

Sleep Problems, Decision-Making, and Suicide Attempts During Adolescence: A Longitudinal Birth Cohort Study

Michaela Pawley^a^, Isabel Morales-Muñoz^b,c^, Andrew Bagshaw^b,d^, Nicole K. Y. Tang^a^

^a^Department of Psychology, University of Warwick, Coventry, UK.

^b^School of Psychology, University of Birmingham, West Midlands, UK.

^c^Institute for Mental Health, University of Birmingham, West Midlands, UK.

^d^Centre for Human Brain Health, University of Birmingham, West Midlands, UK.

Correspondence to Michaela Pawley, 6 University Road, Coventry, Warwickshire, email: michaela.pawley@warwick.ac.uk

**Supplementary Material**

**Methods**

***Measures***

**Total Time in Bed.** For bedtime, response choices were recoded as the following: “Before 9pm,” (recoded as 8:30), “9-9:59pm,” (9:30), “10-10:59pm,” (10:30), “11-midnight,” (11:30), “After midnight” (12:30). Wake-up response choices for school days were coded as: “Before 6am,” (5:30), “6-6:59am,” (6:30), “7-7:59am” (7:30), “8-8:59am” (8:30) and “After 9 am” (9:30). Wake-up response choices for non-school days were recoded as: “Before 8am” (7:30), “8-8:59am” (8:30), “9-9:59am” (9:30), “10-10:59am” (10:30), “11-11:59am” (11:30), and “After midday” (12:30). To then generate values for total time in bed, the difference was taken between the recoded values corresponding to the same time period (e.g., wake-up time on school night – bedtime on school night).

**Social Jetlag.** Wittman’s original formula [1] was used to assess social jetlag, which represents a misalignment between one’s circadian clock and social clock. To create this, the midpoint between the onset and offset of sleep was taken on school days (MSW) and non-school/free days (MSF).

**Sleep Onset Latency.** Categorical options were recoded as “0-15 minutes” (7.5 min), “16-30 minutes” (23 min), “31-45 minutes” (38 min), “46-60 minutes” (53 min), and “More than 60 minutes” (90 min). This method of recoding mirrors previous research utilising this measure. [2]

**Night Awakenings.** Again, original categorical response choices were recoded as “1 = All of the time” (5), “2 = Most of the time” (4), “3 = A good bit of the time” (3), “4 = Some of the time” (2), “5 = A little of the time” (1), and “6 = None of the time” (0). This inversion of the original scale was done so higher scores reflected more interrupted sleep to be consistent with the other sleep measures.

**Rational Decision-Making**. This is measured by the proportion of trials where the participant bet on the most likely outcome (a value of one reflects all trials involving the selection of the most likely colour and zero indicating least likely colour). This dependent variable was chosen based on representing participants’ tendency to make the optimum decision given the explicit payoff and probability for each colour choice. Poor rational decision-making assessed with this specific measure has been associated with adolescent lifetime and repetitive non-suicidal self-injury [3] and suicidality. [4]

**Overall Proportion Bet.** This outcome measure is assessed by the mean proportion of points bet across trials and is considered a measure of risk-sensitive decision-making that is associated with adolescent suicide attempts. [5] Scores are limited to 0.05 and 0.95 with higher values reflecting riskier betting patterns.

**Risk-Taking**. This variable is calculated as the mean proportion of points bet on trials when the optimal colour was selected. Percentages range from 5% to 95% with higher values representing higher reward-sensitivity or lower punishment-sensitivity. Lower scores have been found in suicidal acute adult psychiatric patients compared with healthy adults. [6]

**Risk Adjustment.** This is measured via the tendency to gamble greater percentages of points on trials when the majority of boxes are of the selected colour. To illustrate, it is expected that participants would bet more on a blue box when the ratio of boxes is 8:2 (blue:red) than 6:4 (blue:red). Therefore, higher scores represent a higher proportion of points bet as the ratio increases. Significant differences have been shown between suicidal acute adult psychiatric patients and healthy adults. [6]

**Deliberation Time.** This is the mean time (in milliseconds) taken to select a box colour response.

**Delay Aversion.** This is calculated as the difference in percentage bet when betting proportions are presented in ascending versus descending order. [7] Participants who are quick or impulsive are more likely to bet larger amounts when the betting options are presented in descending order. Scores are distributed from -0.9 to 0.9, with higher values indicating greater impulsivity.

**Ethnicity**. Ethnicity was initially recorded as 11 categories: White (N = 8964; 79.6%), Mixed (N = 534; 4.7%), Indian (N = 303; 2.7%), Pakistani (N = 574; 5.1%), Bangladeshi (N = 252; 2.2%), Other Asian (N = 100; 0.9%), Black Caribbean (N = 116; 1.0%), Black African (N = 216; 1.9%), Other Black (N = 31; 0.3%), Chinese (N = 21; 0.2%), and Other Ethnic group (N = 156; 1.4%).

**Socioeconomic Status.** Net household income was provided by the main parent or caregiver in banded responses of take-home income after tax and other deductions in additions to sources of income implicitly including state benefits. The MCS then generated a continuous measure of income utilising interval regression techniques, with the process outlined in the data documentation. [8] This was equivalised according to the Organisation for Economic Co-operation and Development (OECD)-modified equivalence scale to account for the size and structure of each household. Families with an equivalised income below 60% of the contemporary national median income were considered to indicate low socioeconomic status.

***Missing Data.***

Adolescents lost to attrition were more frequently males, from an ethnic minority background, lower family income, with greater birth weight.

**Table S1**

*Associations Between Various Variables Possibly Related to Attrition and Participation At 17 Years of Age*

| Participation at 17 years | | | |
| --- | --- | --- | --- |
|  | β | *P* value | OR (95% CI) |
| Birth Weight | 0.076 | 0.002* | 1.079 (1.028, 1.134) |
| Labour Complications | 0.057 | 0.076 | 1.059 (0.994, 1.128) |
| Ethnicity | -0.141 | <0.001* | 0.868 (0.804, 0.937) |
| Sex | -0.212 | <0.001* | 0.809 (0.764, 0.857) |
| Family income | -0.545 | <0.001* | 0.580 (0.546, 0.616) |
| Have close friends, 14 years | 0.037 | 0.792 | 1.038 (0.787, 1.368) |
| Longstanding Illness | -0.095 | 0.126 | 0.910 (0.806, 1.027) |
| Depressive symptoms, 14 years | 0.006 | 0.193 | 1.006 (0.997, 1.014) |

Logistic regression results with participation as an outcome and variables that have been related to systematic attrition bias as predictors. Birth weight, ethnicity, sex, and family income were significantly associated with participation at 17 years of age.

**p*<0.05

**Results**

**Table S2**

*Participant Sociodemographic, Clinical, Sleep, Risk-Taking and Decision-Making Characteristics in Waves Six and Seven*

|  | Wave Six (14 years) (N=11717) | | | Wave Seven (17 years) (N=10201) | | |
| --- | --- | --- | --- | --- | --- | --- |
| Sociodemographic Characteristics | N | Frequency | % | N | Frequency | % |
| Sex (Male/Female) | 11717 | 5879/5838 | 50.2/49.8 | 10201 | 4995/5206 | 49.0/51.0 |
| Ethnicity (White/Other) | 11267 | 8964/2303 | 79.6/20.4 | 10172 | 8228/1944 | 80.9/19.1 |
| Family Income (High/Low) | 11705 | 8245/3460 | 70.4/29.6 | 10175 | 6965/3210 | 68.5/31.5 |
| Suicide Attempt and Self-Harm | N | Frequency / Mean | % / SD | N | Frequency / Mean | % / SD |
| Suicide Attempt (Yes/No) | NA | | | 9723 | 721/9002 | 7.4/92.6 |
| Self-Harm (Yes/No) | 11145 | 1634/9511 | 14.7/85.3 | 9631 | 2203/7428 | 22.9/77.1 |
| Other Variables | N | Frequency / Mean | % / SD | N | Frequency / Mean | % / SD |
| Currently Smoking (Yes/No) | 11146 | 218/10928 | 2.0/98.0 | 9943 | 1120/8823 | 11.3/88.7 |
| Self-esteem (Score range 0 to 15) | 11012 | 10.57 | 2.89 | 9947 | 10.02 | 3.20 |
| Emotional Symptoms (Score range 0 to 10) | 11329 | 2.05 | 2.14 | 9742 | 3.49 | 2.46 |
| Conduct Problems (Score range 0 to 10) | 11331 | 1.42 | 1.63 | 9742 | 1.68 | 1.52 |
| Hyperactivity/Inattention (Score range 0 to 10) | 11324 | 2.99 | 2.40 | 9741 | 3.95 | 2.32 |
| Peer Problems (Score range 0 to 10) | 11334 | 1.74 | 1.82 | 9741 | 2.15 | 1.72 |
| Depressive Symptoms (Score range 0 to 26) | 11045 | 5.52 | 5.86 | NA | | |
| Sleep Variables | N | Frequency / Mean | % / SD | N | Frequency / Mean | % / SD |
| Bedtime, school day (Before 9pm/9-9:59pm/10-10:59pm/11-midnight/After midnight) | 11324 | 529/3257/4574/2271/693 | 4.7/28.8/40.4/20.1/6.1 | NA | | |
| Wake-up time, school day (Before 6am/6-6:59am/7-7:59am/8-8:59am/After 9am) | 11336 | 439/4539/5913/374/71 | 3.9/40.0/52.2/3.3/0.6 | NA | | |
| *Total time in bed, school day (Score range 5 to 13 hours) | 11319 | 8.62 | 1.05 | NA | | |
| Bedtime, non-school day (Before 9pm/9-9:59pm/10-10:59pm/11-midnight/After midnight) | 11327 | 96/614/2680/4199/3738 | 0.8/5.4/23.7/37.1/33.0 | NA | | |
| Wake-up time, school day (Before 8am/8-8:59am/9-9:59am/10-10:59am/11-11:59am/After midday) | 11323 | 870/1755/2854/3355/1749/740 | 7.7/15.5/25.2/29.6/15.4/6.5 | NA | | |
| *Total time in bed, non-school day (Score range 7 to 16 hours) | 11312 | 10.53 | 1.23 | NA | | |
| *Social Jetlag (Score range -2 to 5.5 hours) | 11300 | 1.97 | 0.84 | NA | | |
| Sleep Onset Latency (0-15 minutes/16-30 minutes/31-45 minutes/46-60 minutes/More than 60 minutes) | 11266 | 3770/3748/1772/867/1109 | 33.5/33.3/15.7/7.7/9.8 | NA | | |
| *Sleep Onset Latency (Score range 7.5 to 90 minutes) | 11266 | 29.08 | 24.29 | NA | | |
| Frequency of Night Awakenings (Score range 0 to 5) | 11302 | 1.38 | 1.37 | NA | | |
| Cambridge Gambling Task Variables | N | Mean | SD | N | Mean | SD |
| Rational Decision-Making (Score range 0.00 to 1.00) | 10710 | 0.88 | 0.13 | NA | | |
| Risk-Taking (Score range 0.05 to 0.95) | 10709 | 0.52 | 0.15 | NA | | |
| Risk Adjustment (Score range -4.06 to 5.28) | 10709 | 0.99 | 0.97 | NA | | |
| Deliberation Time (Score range 362 to 23691 milliseconds) | 10710 | 2338.76 | 946.61 | NA | | |
| Delay Aversion (Score range -0.90 to 0.90) | 10705 | 0.27 | 0.22 | NA | | |
| Overall Proportion Bet (Score range 0.05 to 0.95) | 10710 | 0.48 | 0.14 | NA | | |

Descriptive and frequency information of the variables of interest of participants from waves 6 and 7 in the Millennium Cohort Study.

N: Sample size; SD: Standard Deviation; %: Proportion of the Sample; NA: Data not available; *: Derived variable.

**Table S3**

*Weighted Unadjusted and Adjusted Interactions between Frequency of Night Awakenings and CGT Measures at 14 Years with Reported Suicide Attempt at 17 Years as the Outcome*

|  | Model A | | | Model B | | |
| --- | --- | --- | --- | --- | --- | --- |
|  | OR | 95% CI | *P* value | OR | OR 95% CI | *P* value |
| Night Awakenings X RDM | 1.98 | 1.35, 2.90 | <0.001** | 2.12 | 1.33, 3.37 | 0.002** |
| Night Awakenings | 0.86 | 0.61, 1.20 | 0.375 | 0.59 | 0.39, 0.90 | 0.013* |
| RDM | 0.11 | 0.04, 0.30 | <0.001** | 0.09 | 0.03, 0.28 | <0.001** |
| Sex | - | - | - | 0.79 | 0.64, 0.97 | 0.022* |
| Socioeconomic Status | - | - | - | 2.16 | 1.74, 2.67 | <0.001** |
| Ethnicity | - | - | - | 1.42 | 1.06, 1.91 | 0.020* |
| Regularly Smokes Cigarettes, 14 years | - | - | - | 0.96 | 0.59, 1.57 | 0.869 |
| Self-Harm, 14 years | - | - | - | 4.34 | 3.47, 5.42 | <0.001** |
| Self-Esteem, 14 years | - | - | - | 0.98 | 0.94, 1.01 | 0.204 |
| Depressive Symptoms, 14 years | - | - | - | 1.07 | 1.05, 1.09 | <0.001** |
| Night Awakenings X DA | 0.86 | 0.66, 1.11 | 0.248 | 0.90 | 0.67, 1.21 | 0.488 |
| Night Awakenings | 1.63 | 1.49, 1.79 | <0.001** | 1.18 | 1.06, 1.32 | 0.002** |
| DA | 1.70 | 0.84, 3.42 | 0.140 | 1.45 | 0.69, 3.07 | 0.330 |
| Sex | - | - | - | 0.78 | 0.64, 0.96 | 0.020* |
| Socioeconomic Status | - | - | - | 2.17 | 1.76, 2.68 | <0.001** |
| Ethnicity | - | - | - | 1.40 | 1.04, 1.89 | 0.025* |
| Regularly Smokes Cigarettes, 14 years | - | - | - | 0.96 | 0.59, 1.57 | 0.871 |
| Self-Harm, 14 years | - | - | - | 4.27 | 3.42, 5.33 | <0.001** |
| Self-Esteem, 14 years | - | - | - | 0.97 | 0.94, 1.01 | 0.162 |
| Depressive Symptoms, 14 years | - | - | - | 1.07 | 1.05, 1.09 | <0.001** |
| Night Awakenings X DT | 1.00 | 1.00, 1.00 | 0.087 | 1.00 | 1.00, 1.00 | 0.077 |
| Night Awakenings | 1.76 | 1.52, 2.03 | <0.001** | 1.32 | 1.12, 1.55 | 0.001** |
| DT | 1.00 | 1.00, 1.00 | <0.001** | 1.00 | 1.00, 1.00 | <0.001** |
| Sex | - | - | - | 0.77 | 0.63, 0.95 | 0.014* |
| Socioeconomic Status | - | - | - | 2.13 | 1.72, 2.64 | <0.001** |
| Ethnicity | - | - | - | 1.41 | 1.05, 1.90 | 0.023* |
| Regularly Smokes Cigarettes, 14 years | - | - | - | 0.99 | 0.61, 1.61 | 0.956 |
| Self-Harm, 14 years | - | - | - | 4.27 | 3.42, 5.33 | <0.001** |
| Self-Esteem, 14 years | - | - | - | 0.97 | 0.94, 1.01 | 0.146 |
| Depressive Symptoms, 14 years | - | - | - | 1.07 | 1.05, 1.09 | <0.001** |
| Night Awakenings X OPB | 0.86 | 0.58, 1.26 | 0.431 | 0.95 | 0.61, 1.47 | 0.812 |
| Night Awakenings | 1.68 | 1.40, 2.02 | <0.001** | 1.18 | 0.96, 1.45 | 0.127 |
| OPB | 0.41 | 0.15, 1.13 | 0.085 | 0.48 | 0.16, 1.46 | 0.192 |
| Sex | - | - | - | 0.83 | 0.67, 1.03 | 0.085 |
| Socioeconomic Status | - | - | - | 2.21 | 1.79, 2.73 | <0.001** |
| Ethnicity | - | - | - | 1.39 | 1.03, 1.87 | 0.030* |
| Regularly Smokes Cigarettes, 14 years | - | - | - | 1.02 | 0.62, 1.66 | 0.952 |
| Self-Harm, 14 years | - | - | - | 4.29 | 3.43, 5.36 | <0.001** |
| Self-Esteem, 14 years | - | - | - | 0.97 | 0.94, 1.01 | 0.159 |
| Depressive Symptoms, 14 years | - | - | - | 1.07 | 1.05, 1.09 | <0.001** |
| Night Awakenings X RA | 1.08 | 1.02, 1.14 | 0.010* | 1.08 | 1.01, 1.15 | 0.025* |
| Night Awakenings | 1.45 | 1.35, 1.56 | <0.001** | 1.07 | 0.97, 1.17 | 0.176 |
| RA | 0.77 | 0.67, 0.89 | <0.001** | 0.80 | 0.68, 0.94 | 0.007** |
| Sex | - | - | - | 0.80 | 0.65, 0.98 | 0.032* |
| Socioeconomic Status | - | - | - | 2.13 | 1.72, 2.64 | <0.001** |
| Ethnicity | - | - | - | 1.42 | 1.05, 1.91 | 0.022* |
| Regularly Smokes Cigarettes, 14 years | - | - | - | 0.96 | 0.59, 1.55 | 0.854 |
| Self-Harm, 14 years | - | - | - | 4.32 | 3.46, 5.40 | <0.001** |
| Self-Esteem, 14 years | - | - | - | 0.97 | 0.94, 1.01 | 0.174 |
| Depressive Symptoms, 14 years | - | - | - | 1.07 | 1.05, 1.09 | <0.001** |
| Night Awakenings X RT | 0.94 | 0.66, 1.36 | 0.756 | 0.99 | 0.66, 1.50 | 0.978 |
| Night Awakenings | 1.61 | 1.33, 1.94 | <0.001** | 1.15 | 0.93, 1.43 | 0.193 |
| RT | 0.32 | 0.12, 0.84 | 0.021* | 0.44 | 0.15, 1.26 | 0.124 |
| Sex | - | - | - | 0.84 | 0.68, 1.04 | 0.102 |
| Socioeconomic Status | - | - | - | 2.21 | 1.78, 2.73 | <0.001** |
| Ethnicity | - | - | - | 1.39 | 1.03, 1.87 | 0.030* |
| Regularly Smokes Cigarettes, 14 years | - | - | - | 1.02 | 0.62, 1.66 | 0.953 |
| Self-Harm, 14 years | - | - | - | 4.29 | 3.44, 5.36 | <0.001** |
| Self-Esteem, 14 years | - | - | - | 0.97 | 0.94, 1.01 | 0.152 |
| Depressive Symptoms, 14 years | - | - | - | 1.07 | 1.05, 1.09 | <0.001** |

Moderation analyses with frequency of night awakenings at 14 years as a predictor, each outcome measure from the Cambridge Gambling Task assessed at 14 years as a moderator and reporting an attempted suicide at 17 years as the dependent variable. After applying the Bonferroni correction for multiple testing, rational decision-making was found to significantly moderate the association between night awakenings and reported suicide attempt in both the unadjusted and adjusted model. In the adjusted model, variables were coded as the following = sex (female [referent] versus male), socioeconomic status (high [referent] versus low), ethnicity (ethnic minority [referent] versus white), regularly smokes cigarettes (no [referent] versus yes), self-harm (no [referent] versus yes), self-esteem (entered as a continuous variable with higher scores indicating good self-esteem), and depressive symptoms (entered as a continuous variable with higher scores indicating greater severity).

OR: Odds Ratio; 95% CI: 95% Confidence Interval. RDM: Rational Decision-Making; DA: Delay Aversion; DT: Deliberation Time; OPB: Overall Proportion Bet; RA: Risk Adjustment; RT: Risk-Taking.

*p<0.05; **p<0.008, Bonferroni corrected for the number of statistical tests undertaken in the table.

**Table S4**

*Weighted Unadjusted and Adjusted Interactions between Total Time in Bed on School Days and CGT Measures at 14 Years with Reported Suicide Attempt at 17 Years as the Outcome*

|  | Model A | | | Model B | | |
| --- | --- | --- | --- | --- | --- | --- |
|  | OR | 95% CI | *P* value | OR | OR 95% CI | *P* value |
| Total Time in Bed School X RDM | 0.98 | 0.57, 1.70 | 0.944 | 1.35 | 0.70, 2.60 | 0.372 |
| Total Time in Bed School | 0.65 | 0.40, 1.05 | 0.080 | 0.66 | 0.37, 1.18 | 0.164 |
| RDM | 0.45 | 0.01, 42.27 | 0.732 | 0.03 | 0.00, 7.43 | 0.214 |
| Sex | - | - | - | 0.77 | 0.63, 0.95 | 0.014* |
| Socioeconomic Status | - | - | - | 2.22 | 1.79, 2.74 | <0.001** |
| Ethnicity | - | - | - | 1.39 | 1.03, 1.86 | 0.031* |
| Regularly Smokes Cigarettes, 14 years | - | - | - | 0.91 | 0.56, 1.49 | 0.709 |
| Self-Harm, 14 years | - | - | - | 4.39 | 3.52, 5.48 | <0.001** |
| Self-Esteem, 14 years | - | - | - | 0.98 | 0.94, 1.02 | 0.227 |
| Depressive Symptoms, 14 years | - | - | - | 1.08 | 1.06, 1.10 | <0.001** |
| Total Time in Bed School X DA | 0.88 | 0.61, 1.27 | 0.497 | 0.87 | 0.58, 1.30 | 0.493 |
| Total Time in Bed School | 0.66 | 0.58, 0.75 | <0.001** | 0.89 | 1.78, 1.02 | 0.099 |
| DA | 4.28 | 0.20, 91.15 | 0.352 | 4.01 | 0.14, 116.81 | 0.419 |
| Sex | - | - | - | 0.78 | 0.63, 0.95 | 0.016* |
| Socioeconomic Status | - | - | - | 2.27 | 1.84, 2.80 | <0.001** |
| Ethnicity | - | - | - | 1.36 | 1.01, 1.83 | 0.040* |
| Regularly Smokes Cigarettes, 14 years | - | - | - | 0.89 | 0.54, 1.46 | 0.644 |
| Self-Harm, 14 years | - | - | - | 4.36 | 3.49, 5.44 | <0.001** |
| Self-Esteem, 14 years | - | - | - | 0.98 | 0.94, 1.01 | 0.218 |
| Depressive Symptoms, 14 years | - | - | - | 1.08 | 1.06, 1.10 | <0.001** |
| Total Time in Bed School X DT | 1.00 | 1.00, 1.00 | 0.222 | 1.00 | 1.00, 1.00 | 0.381 |
| Total Time in Bed School | 0.58 | 0.48, 0.69 | <0.001** | 0.93 | 0.76, 1.15 | 0.500 |
| DT | 1.00 | 1.00, 1.00 | 0.487 | 1.00 | 1.00, 1.00 | 0.160 |
| Sex | - | - | - | 0.77 | 0.62, 0.94 | 0.012* |
| Socioeconomic Status | - | - | - | 2.21 | 1.79, 2.73 | <0.001** |
| Ethnicity | - | - | - | 1.38 | 1.02, 1.85 | 0.036* |
| Regularly Smokes Cigarettes, 14 years | - | - | - | 0.93 | 0.57, 1.52 | 0.773 |
| Self-Harm, 14 years | - | - | - | 4.37 | 3.50, 5.46 | <0.001** |
| Self-Esteem, 14 years | - | - | - | 0.97 | 0.94, 1.01 | 0.167 |
| Depressive Symptoms, 14 years | - | - | - | 1.08 | 1.06, 1.10 | <0.001** |
| Total Time in Bed School X OPB | 1.89 | 1.09, 3.28 | 0.023* | 1.72 | 0.94, 3.13 | 0.078 |
| Total Time in Bed School | 0.47 | 0.37, 0.62 | <0.001** | 0.67 | 0.50, 0.90 | 0.007** |
| OPB | 0.00 | 0.00, 0.15 | 0.006** | 0.01 | 0.00, 0.79 | 0.040* |
| Sex | - | - | - | 0.81 | 0.66, 1.01 | 0.056 |
| Socioeconomic Status | - | - | - | 2.29 | 1.86, 2.83 | <0.001** |
| Ethnicity | - | - | - | 1.35 | 1.01, 1.82 | 0.045* |
| Regularly Smokes Cigarettes, 14 years | - | - | - | 0.97 | 0.59, 1.58 | 0.888 |
| Self-Harm, 14 years | - | - | - | 4.34 | 3.48, 5.42 | <0.001** |
| Self-Esteem, 14 years | - | - | - | 0.98 | 0.94, 1.01 | 0.206 |
| Depressive Symptoms, 14 years | - | - | - | 1.08 | 1.06, 1.10 | <0.001** |
| Total Time in Bed School X RA | 0.96 | 0.89, 1.04 | 0.328 | 0.98 | 0.89, 1.08 | 0.658 |
| Total Time in Bed School | 0.66 | 0.60, 0.74 | <0.001** | 0.88 | 0.77, 0.99 | 0.033* |
| RA | 1.20 | 0.61, 2.36 | 0.595 | 1.10 | 0.50, 2.41 | 0.819 |
| Sex | - | - | - | 1.79 | 0.64, 0.97 | 0.025* |
| Socioeconomic Status | - | - | - | 2.21 | 1.79, 2.74 | <0.001** |
| Ethnicity | - | - | - | 1.37 | 1.02, 1.85 | 0.036* |
| Regularly Smokes Cigarettes, 14 years | - | - | - | 0.90 | 0.55, 1.47 | 0.668 |
| Self-Harm, 14 years | - | - | - | 4.39 | 3.52, 5.48 | <0.001** |
| Self-Esteem, 14 years | - | - | - | 0.98 | 0.94, 1.01 | 0.206 |
| Depressive Symptoms, 14 years | - | - | - | 1.08 | 1.06, 1.10 | <0.001** |
| Total Time in Bed School X RT | 1.69 | 1.01, 2.83 | 0.046* | 1.59 | 0.91, 2.80 | 0.106 |
| Total Time in Bed School | 0.49 | 0.38, 0.64 | <0.001** | 0.68 | 0.51, 0.92 | 0.011* |
| RT | 0.00 | 0.00, 0.27 | 0.011* | 0.01 | 0.00, 1.07 | 0.053 |
| Sex | - | - | - | 0.82 | 0.66, 1.02 | 0.068 |
| Socioeconomic Status | - | - | - | 2.29 | 1.86, 2.83 | <0.001** |
| Ethnicity | - | - | - | 1.35 | 1.01, 1.82 | 0.046* |
| Regularly Smokes Cigarettes, 14 years | - | - | - | 0.96 | 0.59, 1.57 | 0.880 |
| Self-Harm, 14 years | - | - | - | 4.34 | 3.48, 5.42 | <0.001** |
| Self-Esteem, 14 years | - | - | - | 0.98 | 0.94, 1.01 | 0.201 |
| Depressive Symptoms, 14 years | - | - | - | 1.08 | 1.06, 1.10 | <0.001** |

Moderation analyses with total time in bed on school days at 14 years as a predictor, each outcome measure from the Cambridge Gambling Task assessed at 14 years as a moderator and reported attempted suicide at 17 years as the dependent variable. After applying the Bonferroni correction for multiple testing, no significant interactions were present in any of the unadjusted or adjusted models.

In the adjusted model, variables were coded as the following = sex (female [referent] versus male), socioeconomic status (high [referent] versus low), ethnicity (ethnic minority [referent] versus white), regularly smokes cigarettes (no [referent] versus yes), self-harm (no [referent] versus yes), self-esteem (entered as a continuous variable with higher scores indicating good self-esteem), and depressive symptoms (entered as a continuous variable with higher scores indicating greater severity).

OR: Odds Ratio; 95% CI: 95% Confidence Interval. RDM: Rational Decision-Making; DA: Delay Aversion; DT: Deliberation Time; OPB: Overall Proportion Bet; RA: Risk Adjustment; RT: Risk-Taking.

*p<0.05; **p<0.008, Bonferroni corrected for the number of statistical tests undertaken in the table.

**References**

1. Wittmann M, Dinich J, Merrow M, Roenneberg T. Social Jetlag: Misalignment of Biological and Social Time. *Chronobiol Int*. 2006;23(1-2):497-509. doi:10.1080/07420520500545979

2. Hisler G, Twenge JM, Krizan Z. Associations between screen time and short sleep duration among adolescents varies by media type: evidence from a cohort study. *Sleep Med*. 2020;66:92-102. doi:10.1016/j.sleep.2019.08.007

3. Lutz NM, Chamberlain SR, Goodyer IM, et al. Behavioral measures of impulsivity and compulsivity in adolescents with nonsuicidal self-injury. *CNS Spectr*. 2022;27(5):604-612. doi:10.1017/S1092852921000274

4. Chamberlain SR, Odlaug BL, Schreiber LRN, Grant JE. Clinical and neurocognitive markers of suicidality in young adults. *J Psychiatr Res*. 2013;47(5):586-591. doi:10.1016/j.jpsychires.2012.12.016

5. Ackerman JP, McBee-Strayer SM, Mendoza K, et al. Risk-Sensitive Decision-Making Deficit in Adolescent Suicide Attempters. *J Child Adolesc Psychopharmacol*. 2015;25(2):109-113. doi:10.1089/cap.2014.0041

6. Reiråskag MA, Brokke SS, Rohde G, Bertelsen TB, Landrø NI, Haaland VØ. Decision-making deficits in suicidal acute psychiatric patients: Insights from the Cambridge Gambling Task. *Cogent Psychol*. 2024;11(1). doi:10.1080/23311908.2024.2433851

7. Rogers RD. Dissociable Deficits in the Decision-Making Cognition of Chronic Amphetamine Abusers, Opiate Abusers, Patients with Focal Damage to Prefrontal Cortex, and Tryptophan-Depleted Normal Volunteers Evidence for Monoaminergic Mechanisms. *Neuropsychopharmacology*. 1999;20(4):322-339. doi:10.1016/S0893-133X(98)00091-8

8. Centre for Longitudinal Studies. *User Guide (Surveys 1-5)*.; 2020. www.cls.ucl.ac.uk.
